# Dataset on the influence of zinc foliar application and vermicompost on agromorphogenic traits of *Aloe vera*

**DOI:** 10.1016/j.dib.2021.107436

**Published:** 2021-09-30

**Authors:** Md. Marufur Rahman, Md. Ashraful Alam, Md. Musfikus Salehin, Golam Sagir Ahammad, Rownoke Jannat Janny, Nusrat Jahan, Md. Maksudul Haque

**Affiliations:** a Regional Station Rangpur, Bangladesh Institute of Research and Training on Applied Nutrition (BIRTAN), Rangpur, Bangladesh; b Plant Breeding Division, Spices Research Center, Bangladesh Agricultural Research Institute (BARI), Shibganj, Bogura, Bangladesh; c Regional Station Netrokona, Bangladesh Institute of Research and Training on Applied Nutrition (BIRTAN), Netrokona, Bangladesh; d Head Office, Bangladesh Institute of Research and Training on Applied Nutrition (BIRTAN), Araihazar, Narayanganj, Bangladesh; e Plant Breeding Division, Bangladesh Rice Research Institute (BRRI), Gazipur, Bangladesh; f Head Office, Bangladesh Institute of Research and Training on Applied Nutrition (BIRTAN), Araihazar, Narayanganj, Bangladesh

**Keywords:** *Aloe vera*, Fertilizer, Nutrient, Vermicompst, Zinc

## Abstract

This article presents dataset on agromorphogenic traits of *Aloe vera* treated by foliar application of Zn (zinc) and vermicompost. Data from yield and yield related characters with morphological traits were collected to assess the effect of vermicompost and Zn. The data showed in this dataset article contained 17 agronomic and morphological traits. The collected data were analyzed using excel, statistix 10.0 and STAR software. The analyzed data presented with the help of ANOVA (analysis of variance), mean comparison, correlation co-efficient and principal component analysis (PCA). Data of microclimate, correlation co-efficient and biplot distribution of principal components were presented graphically. The aim of the article is to ensure the data easily accessible and as a brief source of agricultural management information for crop development and production for researcher.

## Specifications Table

**Table:** 

Subject	Agriculture
Specific subject area	Agronomy
Type of data	Table and Figure
How data were acquired	Observation were recorded by measurement in the net house and after harvesting
Data format	Analyzed
Parameters for data collection	Plant height (cm), Number of leaves, Leaf length (cm), Leaf breadth (cm), Number of sucker, Largest leaf length (cm), Largest leaf breadth (cm), Largest leaf weight (g), Single mature leaf weight(g),Total leaf weight (g), Number of tiller, Tiller height (cm), Tiller weight(g), Sucker height (cm), Sucker weight (g), Number of lateral root, Length of tap root(cm)
Description of data collection	Data on 17 agronomic and morphological characters were collected by measuring from 6 month aged plants. Total 3 plants were selected for data collection from each replications. Number of leaves, number of sucker, number of tiller and number of lateral roots were collected and counted by hands. The data of largest leaf length, breadth and weight were measured from the selected plants. The plant height, tiller height, sucker height, length of tap root, leaf length, leaf breadth, single mature leaf weight, total leaf weight, tiller weight and sucker weight were measured and calculated average.
Data source location	*Institution:* Bangladesh Institute of research and Training on Applied Nutrition (BIRTAN), Regional Station, Rangpur, Bangladesh *City:* Rangpur *Country:* Bangladesh *Latitude and longitude (and GPS coordinates, if possible) for collected samples/data:* 25.7439° N and 89.2752° E
Data accessibility	With the article

## Value of the Data

• The data provide the primary effect of Zn and vermicompost on growth, yield and yield related traits of *Aloe vera*.
• The dataset give basic information for researchers to develop management practise for *Aloe vera* production in this climatic region and optimization of fertilizer dose for *Aloe vera* cultivation.
• Data can be used to develop adaptation strategies for *Aloe vera* as a new field crop in the northern region of Bangladesh.

## 1 Data Description

### 1.1 Micro-climate data

**Fig. 1:**
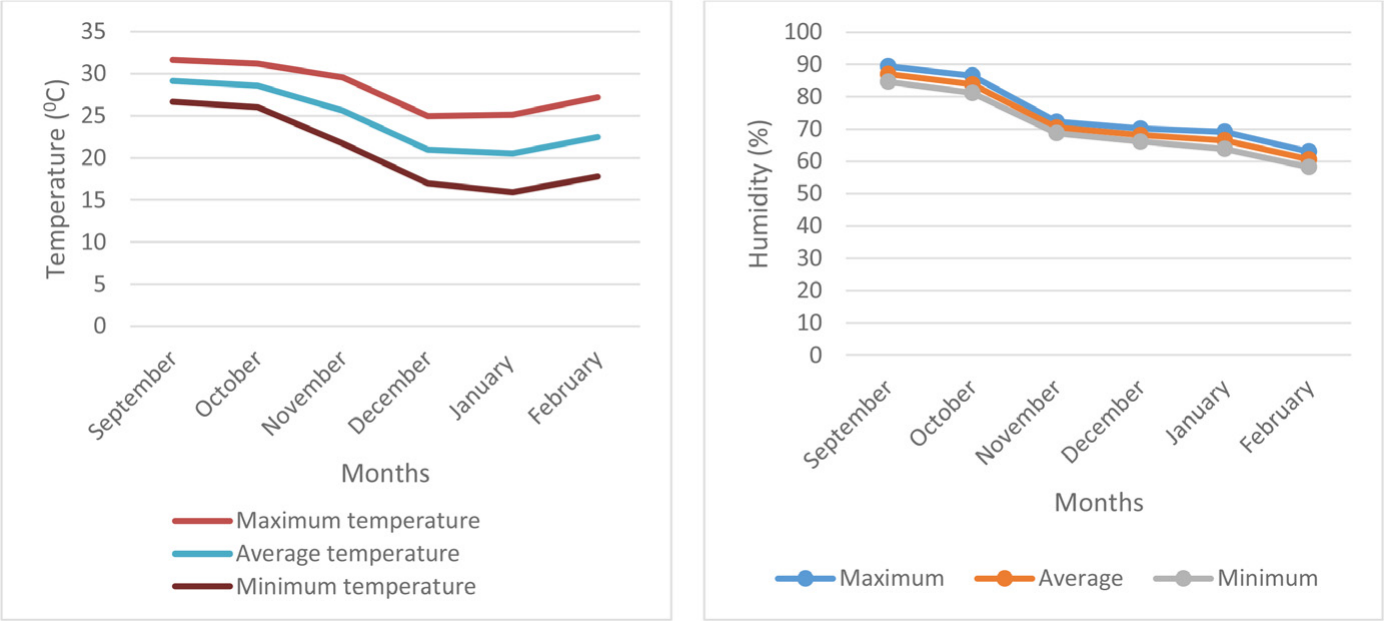
Micro-climate data (temperature and humidity) during *Aloe vera* growing period.

Maximum, minimum and average temperature with humidity during experimental period were presented in Fig. 1. The raw data for micro-climate is presented in the supplementary file “Raw data of micro-climate”.

### 1.2 The effect of Zn foliar application and vermicompost on agromorphogenic traits of *Aloe vera*

Table 1. presents the data on analysis of variance (ANOVA) of nine agronomic traits (Plant height, number of leaves, leaf length, leaf breadth, largest leaf length, largest leaf breadth, single mature leaf weight, largest leaf weight, total leaf weight per plant) of *Aloe vera*. Mean comparison of different treatment and interaction between Zn and vermicompost on nine agronomic traits of *Aloe vera* showed in Tables 3 and 5. The raw data for nine agronomic traits (Tables 1, 3, and 5) is presented in the supplementary file “Raw data of nine agronomic traits”. Analysis of variance (ANOVA) of eight morphological traits (Number of sucker, number of tiller, tiller height, tiller weight, sucker height, sucker weight, number of lateral root and length of tap root) of *Aloe vera* are shown in Table 2. Mean comparison of different treatment and interaction between Zn and vermicompost on eight morphological traits of *Aloe vera* are presented in Tables 4 and 6. The raw data for (Tables 2, 4 and 6) morphological traits is presented in the supplementary file “Raw data of eight morphological traits”.

**Table 1:** Analysis of variance (ANOVA) of nine agronomic traits of *Aloe vera* where (*n* = 3).

		Mean sum of square								
Source of variations	df	PH (cm)	NL (cm)	LL (cm)	LB (cm)	LLL (cm)	LLB (cm)	SMLW (g)	LLW (g)	TLWP (g)
Replication	2	7.36 ns	0.06 ns	3.68 ns	0.04 ns	6.48 ns	0.04 ns	0.74 ns	0.064 ns	153 ns
Zinc	3	102.63**	29.80**	160.86**	4.79**	159.73**	4.73**	593.63**	600.41**	185599**
Vermicompost	3	850.37**	285.69**	188.67**	3.08**	198.67**	3.03**	215.82**	219.62**	336906**
Zinc X Vermicompost	9	19.74**	3.35 ns	6.43**	0.18 ns	7.01**	0.18**	26.46**	28.33**	7779**
Error	30	2.74	1.62	0.94	0.12	1.01	0.12	4.15	4.35	2433
Grand Mean	-	18.53	12.18	15.96	3.50	16.40	3.55	25.96	26.96	334.73
CV%	-	8.93	10.44	6.05	9.75	6.15	10.09	7.85	7.73	14.74

*Keys to abbreviations;* PH: Plant height, NL: Number of leaves, LL: Leaf length, LB: Leaf breadth, LLL: Largest leaf length, LLB: Largest leaf breadth, SMLW: Single mature leaf weight, LLW: Largest leaf weight, TLWP: Total leaf weight per plant, CV: Coefficient of variation, ns: Non-significant, **: significant at the 5% levels of probability.

**Table 2:** Analysis of variance (ANOVA) of eight morphological traits of *Aloe vera* where (*n* = 3).

		Mean sum of square							
Source of variations	df	NS	NT	TH (cm)	TW (g)	SH (cm)	SW (g)	NLR	LTR (cm)
Replication	2	4.02 ns	2.46 ns	20.47 ns	40.83 ns	6.98 ns	290.00 ns	2.25 ns	2.4 ns
Zinc	3	104.44**	64.20**	228.45**	391.84**	247.53**	358.59**	322.99**	5.85**
Vermicompost	3	102.68**	53.63**	33.32**	272.17**	193.22**	361.44**	403.62**	2.26**
Zinc X Vermicompost	9	5.00**	5.00**	20.10**	15.62 ns	14.36 ns	16.05 ns	31.60**	0.28 ns
Error	30	0.63	2.24	7.08	20.64	11.85	21.82	1.96	0.14
Grand mean	-	7.02	5.01	10.21	12.92	15.34	20.26	18.72	3.30
CV%	-	11.33	29.89	26.04	35.16	22.44	23.05	7.48	11.48

*Keys to abbreviations*; NS: Number of sucker, NT: Number of tiller, TH: Tiller height, TW: Tiller weight, SH: Sucker height, SW: Sucker weight, NLR: Number of lateral root, LTR: Length of tap root, CV: Coefficient of variation, ns: Non-significant, **: significant at the 5% levels of probability.

**Fig. 2:**
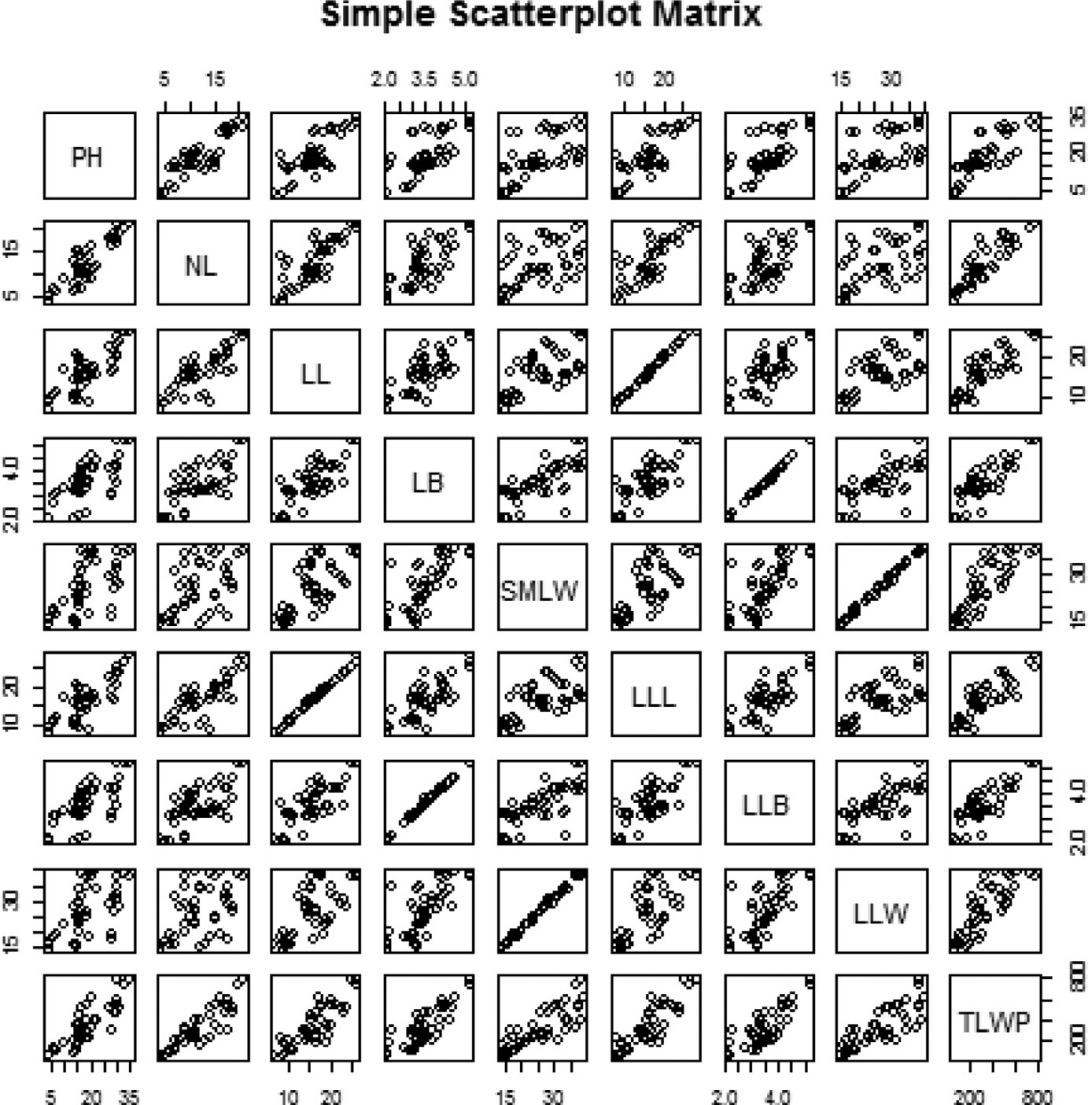
Correlation analysis of nine agronomic traits. *Keys to abbreviations;* PH: Plant height, NL: Number of leaves, LL: Leaf length, LB: Leaf breadth, LLL: Largest leaf length, LLB: Largest leaf breadth, SMLW: Single mature leaf weight, LLW: Largest leaf weight, TLWP: Total leaf weight per plant, Pearson's product-moment correlation, Prob > |r|.

**Fig. 3:**
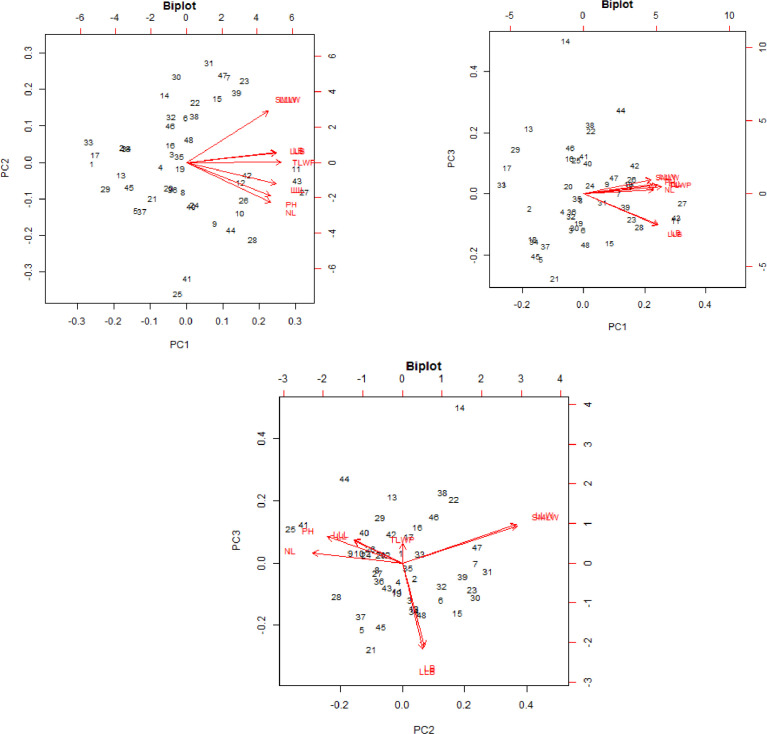
Biplot distribution of agronomic traits of *Aloe vera.*

**Fig. 4:**
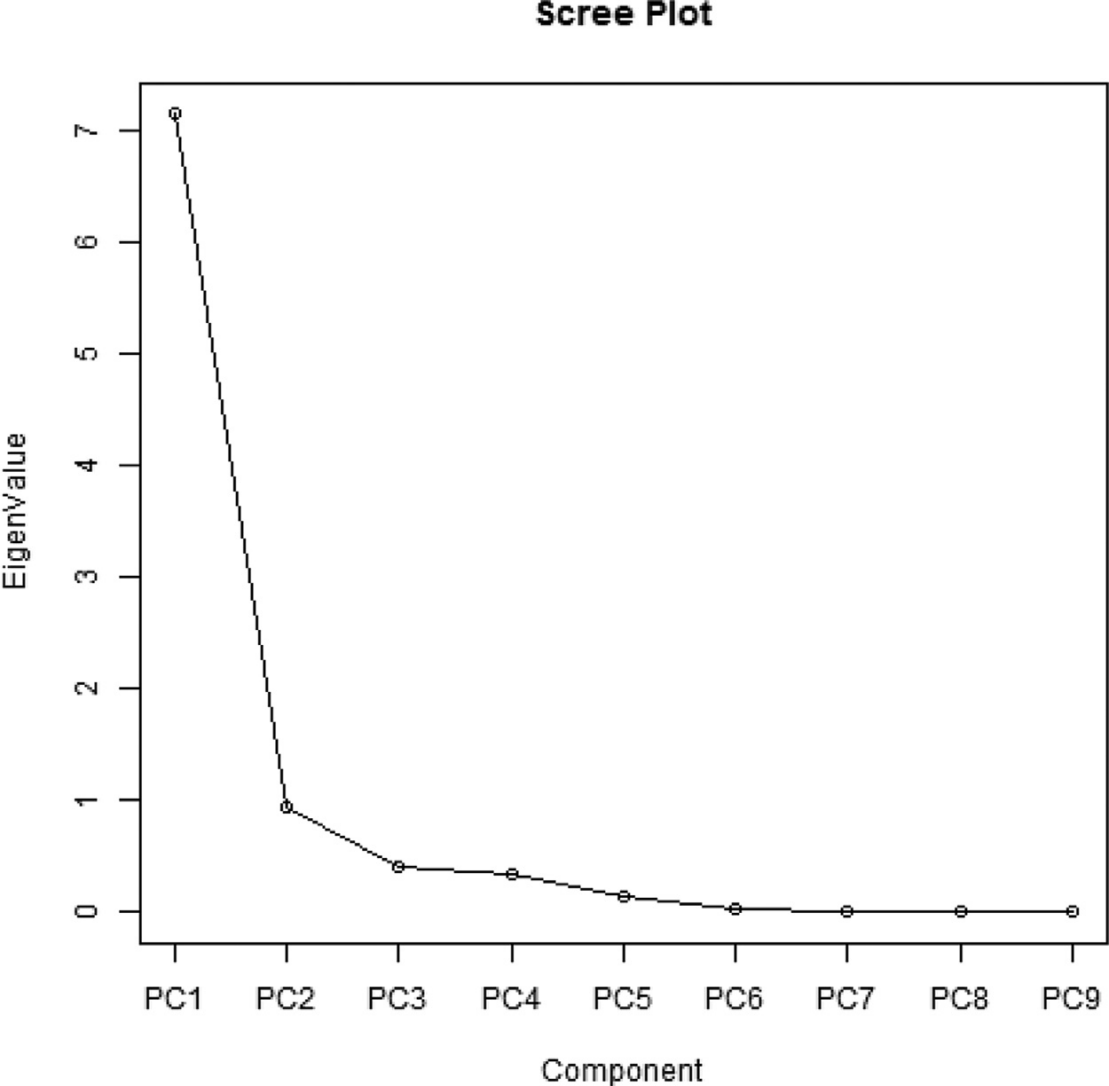
Scree plot of eigenvalue and principal components.

Data on agronomic and morphological traits were collected to investigate the effect of Zn and vermicompost (Tables 3 and 4) and the combination of treatments effect (Tables 5 and 6). Correlation analysis of nine agronomic traits are depicted in Fig. 2. The raw data for correlation analysis and analyzed data of correlation co-efficient is presented in the supplementary file “correlation analysis”. Table 7, shows the principal component analysis of agronomic traits of *Aloe vera*. Table 8, presents eigenvector of agronomic traits of *Aloe vera*. The principal components are most affected the different agronomic trait is presented in Table 9. Biplot distribution to represent the scores of the traits on the principal components of agronomic traits of *Aloe vera* are shown in Figs. 3 and 4. Presents the scree plot of eigenvalue and principal component. The raw data for principal component analysis (Tables 7–9, Figs. 3 and 4) is presented in the supplementary file “principal component analysis”. Phenotype of *Aloe vera* after application of different treatment is presented by using photograph in supplementary file “photograph of *Aloe vera*.”

**Table 3:** Mean comparison of different treatment on nine agronomic traits of *Aloe vera* where (*n* = 3).

	Studied characters									
Treatment	Level	PH (cm)	NL	LL (cm)	LB (cm)	LLL (cm)	LLB (cm)	SMLW (g)	LLW (g)	TLWP (g)
Zinc	Z0	15.79c	10.58c	11.10c	2.78c	11.53c	2.83c	16.97d	17.81d	189.79c
	Z1	17.13bc	11.33bc	15.19b	3.36b	15.69b	3.43b	27.91b	28.84b	332.17b
	Z2	22.55a	14.17a	18.88a	4.31a	19.33a	4.36a	33.89a	34.86a	493.65a
	Z3	18.68b	12.67b	18.70a	3.58b	19.07a	3.61b	25.06c	26.34c	323.32b
Vermicompost	V0	10.34c	7.75c	13.01c	2.96c	13.28d	3.03d	19.77b	20.73c	161.88d
	V1	16.39b	13.5b	15.47b	3.64b	15.86b	3.66b	27.16a	28.12b	371.38b
	V2	30.32a	18.5a	21.71a	4.14a	22.29a	4.21a	29.41a	30.43a	550.7a
	V3	17.10b	9c	13.69c	3.29bc	14.18c	3.33c	27.48a	28.58b	254.97c
LSD_(0.05)_		1.37	1.06	0.8	0.28	0.84	0.29	1.69	1.73	41.25

*Keys to abbreviations*; PH: Plant height, NL: Number of leaves, LL: Leaf length, LB: Leaf breadth, LLL: Largest leaf length, LLB: Largest leaf breadth, SMLW: Single mature leaf weight, LLW: Largest leaf weight, TLWP: Total leaf weight per plant, in each column, different letters are significantly different (*P* < 0.05) by the Tukey HSD All-Pairwise Comparisons Test, LSD: Least significant difference.

**Table 4:** Mean comparison of different treatment on eight morphological traits of *Aloe vera* where (*n* = 3).

	Studied characters								
Treatment	Level	NS	NT	TH (cm)	TW (g)	SH (cm)	SW (g)	NLR	LTR (cm)
Zinc	Z0	3.22d	2.89c	5.76c	5.69c	9.68c	13.51c	12.03d	2.35c
	Z1	6.61c	4.53b	9.84b	12.61b	15.03b	20.47b	17.42c	3.46b
	Z2	10.28a	8.31a	16.20a	19.61a	20.76a	26.90a	23.78a	4.02a
	Z3	7.97b	4.33b	9.08b	13.78b	15.89b	20.17b	21.67b	3.41b
Vermicompost	V0	3.25c	2.39c	7.93b	7.73c	10.23c	16.00b	11.08c	3.11b
	V1	7.33b	5.22b	10.38a	10.79bc	14.16b	18.41b	17.44b	2.81b
	V2	10.39a	7.56a	11.91a	18.80a	19.17a	28.32a	23.94a	3.51a
	V3	7.11b	4.89b	10.66a	14.36b	17.81ab	18.33b	22.42a	3.79a
LSD_(0.05)_		0.66	1.24	2.21	3.78	2.87	3.89	1.16	0.31

*Keys to abbreviations*; NS: Number of sucker, NT: Number of tiller, TH: Tiller height, TW: Tiller weight, SH: Sucker height, SW: Sucker weight, NLR: Number of lateral root, LTR: Length of tap root, in each column, different letters are significantly different (*P* < 0.05) by the Tukey HSD All-Pairwise Comparisons Test, LSD: Least significant difference.

**Table 5:** Mean comparison of interaction between Zn and vermicompost on nine agronomic traits of *Aloe vera.*

Treatment	PH(cm)	NL	LL(cm)	LB(cm)	LLL(cm)	LLB(cm)	SMLW(g)	LLW(g)	TLWP(g)
Z0V0	3.83d	4.33h	8.22g	2.10g	8.63h	2.17h	14.99h	15.85h	65.19g
Z0V1	15.80c	13.00cde	9.41g	3.31cdef	9.87gh	3.33ef	15.87gh	16.72h	207.24efg
Z0V2	28.80a	17.67ab	17.10cd	3.26cdef	17.77d	3.33ef	20.35efgh	21.24fg	362.25cd
Z0V3	14.73c	7.33fgh	9.68g	2.46fg	9.83gh	2.50gh	16.64gh	17.43h	124.48fg
Z1V0	6.50d	6.67gh	10.72fg	2.89efg	11.03g	3.00fg	18.17fgh	19.05gh	121.3fg
Z1V1	16.20c	12.33cde	15.04de	3.42bcdef	15.50ef	3.47ef	31.62abc	32.60b	391.89cd
Z1V2	29.03a	18.00ab	21.36b	4.09bc	21.77bc	4.20bc	31.05bc	31.85b	558.86b
Z1V3	16.77bc	8.33fg	13.66ef	3.03defg	14.47f	3.07efg	30.81bc	31.86b	256.63def
Z2V0	17.50bc	10.33defg	15.62de	3.53bcde	15.77ef	3.63cde	24.50de	25.29de	253.61def
Z2V1	18.37bc	15.00bc	17.62cd	4.38ab	17.93d	4.40b	37.69a	38.80a	565.67b
Z2V2	32.93a	20.67a	25.97a	5.16a	26.90a	5.20a	37.35a	38.27a	772.15a
Z2V3	21.40b	10.67def	16.30de	4.17abc	16.70de	4.20bc	36.02ab	37.10a	383.17cd
Z3V0	13.53c	9.67efg	17.49cd	3.30cdef	17.70d	3.33ef	21.43efg	22.71ef	207.41efg
Z3V1	15.20c	13.67cd	19.79bc	3.43bcdef	20.13c	3.43ef	23.45def	24.37ef	320.74de
Z3V2	30.50a	17.67ab	22.42b	4.07bcd	22.73b	4.10bcd	28.91cd	30.38bc	509.54bc
Z3V3	15.50c	9.67efg	15.11de	3.51bcde	15.70ef	3.57def	26.45cde	27.91cd	255.6def
LSD_(0.05)_	2.75	2.12	1.61	0.57	1.68	0.59	3.39	3.47	82.25

*Keys to abbreviations;* PH: Plant height, NL: Number of leaves, LL: Leaf length, LB: Leaf breadth, LLL: Largest leaf length, LLB: Largest leaf breadth, SMLW: Single mature leaf weight, LLW: Largest leaf weight, TLWP: Total leaf weight per plant, in each column, different letters are significantly different (*P* < 0.05) by the Tukey HSD All-Pairwise Comparisons Test, LSD: Least significant difference.

**Table 6:** Mean comparison of interaction between zinc and vermicompost on eight morphological traits of *Aloe vera.*

Treatment	NS	NT	TH(cm)	TW(g)	SH(cm)	SW(g)	NLR	LTR(cm)
Z0V0	1.44h	1.67f	3.04f	1.81g	4.18g	9.34gh	8.00j	2.50ef
Z0V1	3.67fgh	2.89def	3.43f	4.05fg	11.38ef	14.29fgh	9.67ij	1.79g
Z0V2	5.11ef	4.00cdef	8.48de	7.38efg	15.13cde	21.77cdef	14.56g	2.17fg
Z0V3	2.67gh	3.00def	8.09de	9.51def	8.02fg	8.64h	15.89fg	2.93de
Z1V0	2.33gh	2.00ef	6.40ef	8.00efg	8.93fg	16.81efg	10.78i	2.92de
Z1V1	6.67de	4.44cde	14.91abc	10.39def	13.12def	16.33efgh	17.56f	3.31cd
Z1V2	11.56b	8.89ab	9.71de	18.34bc	18.76bcd	30.57ab	17.89f	3.83abc
Z1V3	5.89def	2.78def	8.32de	13.70cde	19.31bc	18.18def	23.44d	3.76abc
Z2V0	5.33ef	4.22cde	15.76ab	12.80cde	15.89cde	20.63cdef	13.78gh	3.72bc
Z2V1	10.89b	9.56a	16.50a	17.01bcd	18.68bcd	23.87bcde	22.22de	3.61bc
Z2V2	14.44a	10.89a	17.44a	26.89a	23.16ab	35.03a	33.22a	4.39a
Z2V3	10.44bc	8.56ab	15.10abc	21.74ab	25.33a	28.06abc	25.89c	4.37a
Z3V0	3.89fg	1.67f	6.50ef	8.32efg	11.90ef	17.20ef	11.78hi	3.31cd
Z3V1	8.11cd	4.00cdef	6.68ef	11.72cde	13.48def	19.14def	20.33e	2.52ef
Z3V2	10.44bc	6.44bc	12.00bcd	22.57ab	19.63abc	25.92bcd	30.11b	3.67bc
Z3V3	9.44bc	5.22cd	11.12cd	12.50cde	18.57bcd	18.43def	24.44cd	4.12ab
LSD_(0.05)_	1.32	2.49	4.43	7.57	5.74	7.78	2.33	0.63

*Keys to abbreviations;* NS: Number of sucker, NT: Number of tiller, TH: Tiller height, TW: Tiller weight, SH: Sucker height, SW: Sucker weight, NLR: Number of lateral root, LTR: Length of tap root, in each column, different letters are significantly different (P < 0.05) by the Tukey HSD All-Pairwise Comparisons Test, LSD: Least significant difference.

**Table 7:** Principal component analysis of agronomic traits of *Aloe vera.*

PRINCIPAL COMPONENT ANALYSIS									
Statistics	PC1	PC2	PC3	PC4	PC5	PC6	PC7	PC8	PC9
Standard deviation	2.6722	0.9708	0.6348	0.5885	0.3713	0.1488	0.0586	0.0491	0.0423
Proportion of Variance	0.7934	0.1047	0.0448	0.0385	0.0153	0.0025	0.0004	0.0003	0.0002
Cumulative Proportion	0.7934	0.8981	0.9429	0.9814	0.9967	0.9991	0.9995	0.9998	1.0000
EigenValues	7.1407	0.9424	0.4030	0.3463	0.1379	0.0221	0.0034	0.0024	0.0018

*Keys to abbreviations*; PC: Principal component.

**Table 8:** Eigenvector of agronomic traits of *Aloe vera.*

Variables	PC1	PC2	PC3	PC4	PC5	PC6	PC7	PC8	PC9
PH	0.3202	-0.3528	0.1885	0.4165	-0.7405	0.1221	0.0085	0.0218	0.0078
NL	0.3219	-0.4197	0.0724	0.3904	0.4811	-0.5724	-0.0034	-0.0586	-0.0032
LL	0.3416	-0.2275	0.16	-0.5535	-0.0079	-0.0098	0.7067	0.0058	-0.0057
LB	0.3439	0.1043	-0.5969	-0.0008	-0.0414	-0.062	-0.0024	0.6506	0.2928
SMLW	0.3123	0.5368	0.2674	0.0681	-0.0402	-0.1432	0.0199	-0.3128	0.6464
LLL	0.3416	-0.2284	0.1679	-0.5495	-0.0186	-0.0047	-0.7069	-0.0078	0.0289
LLB	0.3421	0.0954	-0.6171	-0.0145	-0.0788	0.0347	-0.0039	-0.6329	-0.2912
LLW	0.3119	0.536	0.2782	0.0449	-0.0609	-0.2266	-0.0173	0.2669	-0.6399
TLWP	0.361	0.0013	0.1398	0.2428	0.4545	0.7618	-0.0006	0.0548	-0.0352

*Keys to abbreviations*; PH: Plant height, NL: Number of leaves, LL: Leaf length, LB: Leaf breadth, LLL: Largest leaf length, LLB: Largest leaf breadth, SMLW: Single mature leaf weight, LLW: Largest leaf weight, TLWP: Total leaf weight per plant, PC: Principal component.

**Table 9:** The different traits affects the principal components.

Traits	PC1	PC2	PC3	PC4	PC5	PC6	PC7	PC8	PC9
PH		0.404	0.718	0.539	0.17				
NL		0.273			-0.959				
LL		0.108	0.37	-0.586				0.711	
LB						-0.695			0.715
SMLW		-0.603	0.298		-0.13		-0.72		
LLL		0.11	0.38	-0.59				-0.701	
LLB						-0.713			-0.699
LLW		-0.612	0.328		-0.181		0.688		
TLWP	0.998								

**Keys to abbreviations**; PH: Plant height, NL: Number of leaves, LL: Leaf length, LB: Leaf breadth, LLL: Largest leaf length, LLB: Largest leaf breadth, SMLW: Single mature leaf weight, LLW: Largest leaf weight, TLWP: Total leaf weight per plant, PC: Principal component.

## 2 Experimental Design, Materials and Methods

### 2.1 Plant production

*Aloe vera* cultivar was collected from local market of Rangpur, Bangladesh. *Aloe vera* plants were grown in nursery bed for sucker production. Around two month old uniform sucker of *Aloe vera* with a size 8 to 10 cm were selected to transfer into plastic pot from nursery bed. The transplanting date was 1 September 2020. The size of plastic pot was 10 inch diameter and 10 inch height with 10 kg soil capacity. Vermicompost were properly mixed with soil prior to transplant of sucker in net house [1]. The experiment was set up in the net house of Bangladesh Institute of Research and Training on Applied Nutrition (BIRTAN), Regional station, Rangpur, Bangladesh in September 2020 to February 2021. Different intercultural operations and standard agronomic practices were performed with the method explained for *Aloe vera* cultivation [2].Observations were recorded after six month of transplanting.

### 2.2 Design of experiment and treatments

The field experiment was conducted in 2020–2021 at Bangladesh Institute of Research and Training on Applied Nutrition, Regional station, Rangpur, Bangladesh. The experimental design was randomized complete block design with 3 replications. Zinc (ZnSO_4_.7H_2_O, Sarker Zinc) was applied as 4 different treatment solutions such as (Control (Z0):0, Z1:1%, Z2:2% and Z3:3%) as foliar application. The vermicompost treatments were (Control (V0):0, V1: 10% vermicompost +90% soil, V2:25% vermicompost+75% soil, V3:50% vermicompost +50% soil) per pot. Zinc sulphate was the source of zinc and zinc solution sprayed at 4 times @ 45 days, 90 days, 135 days and 165 days after transplanting. In treatment Z0 and V0 where no application of Zinc and vermicompost was considered as control treatment. Vermicompost was purchase from the local market (Brand name kulsum vermicompost). Vermicompost was prepared using cow dung with the help of earthworm red wigglers (*Eisenia fetida*).

### 2.3 Data collection

Agronomic characters like (PH: plant height, NL: number of leaves, LL: leaf length, LB: leaf breadth, LLL: largest leaf length, LLB: largest leaf breadth, SMLW: single mature leaf weight, LLW: largest leaf weight, TLWP: total leaf weight per plant) and morphological traits (NS: number of sucker, NT: number of tiller, TH: tiller height, SH: sucker height, SW: sucker weight, NLR: number of lateral root, LTR: length of tap root) were measured [3,4]. Average data of plant characters were recorded from three plants of each replications after six month of transplanting. Leaf harvesting and data collection date was 28 February 2021.

### 2.4 Statistical analysis

Statistix 10.0 software was used to preformed an analysis of variance (ANOVA) at *P* < 0.05; mean values differentiating by Tukey HSD All-Pairwise Comparisons test at *P* < 0.05. Correlation co-efficient analysis and principal component analysis were conducted by Statistical tool for Agricultural Research (STAR) software, version 2.0.1 (2014). Figure on micro climatic data were drawn by EXCEL software.

## CRediT authorship contribution statement

**Md. Marufur Rahman:** Conceptualization, Methodology, Visualization, Data curation, Formal analysis, Writing – original draft. **Md. Ashraful Alam:** Validation, Investigation, Writing – review & editing. **Md. Musfikus Salehin:** Data curation. **Golam Sagir Ahammad:** Data curation. **Rownoke Jannat Janny:** Data curation. **Nusrat Jahan:** Writing – review & editing. **Md. Maksudul Haque:** Visualization, Investigation.

## Declaration of Competing Interest

The authors declare that they have no known competing financial interests or personal rela- tionships that have, or could be perceived to have, influenced the work reported in this article.

## Appendix Supplementary materials

Image, application 1

Image, application 2

Image, application 3

Image, application 4

Image, application 5

Image, application 6

Image, application 7

Image, application 8

Image, application 9

Image, application 10

Image, application 11

Image, application 12

Image, application 13

Image, application 14

Image, application 15

Image, application 16

Image, application 17

Image, application 18

Image, application 19

Image, application 20

Image, application 21

Image, application 22

Image, application 23

Image, application 24

Image, application 25

Image, application 26
