# Supplementary material for: Dataset on the influence of zinc foliar application and vermicompost on agromorphogenic traits of Aloe vera
Source: Data Brief. 2021 Sep 30;38:107436. doi: 10.1016/j.dib.2021.107436 (PMC8498230; doi:10.1016/j.dib.2021.107436)
Supplement: Supplementary file 19 [file mmc19.zip › Table of correlation analysis.docx]

|  | PH | NL | LL | LB | SMLW | LLL | LLB | LLW | TLWP |  |
| --- | --- | --- | --- | --- | --- | --- | --- | --- | --- | --- |
| PH | coef | 1 | 0.8868 | 0.7899 | 0.7105 | 0.5695 | 0.7972 | 0.4657 | 0.4161 | 0.8263 |
|  | p-value | 0 | 0 | 0 | 0 | 0 | 0.0009 | 0.0033 | 0 |  |
|  | n | 48 | 48 | 48 | 48 | 48 | 48 | 48 | 48 | 48 |
| NL | coef | 0.8868 | 1 | 0.8045 | 0.7298 | 0.5217 | 0.8014 | 0.6068 | 0.4655 | 0.8866 |
|  | p-value | 0 | 0 | 0 | 0.0001 | 0 | 0 | 0.0009 | 0 |  |
|  | n | 48 | 48 | 48 | 48 | 48 | 48 | 48 | 48 | 48 |
| LL | coef | 0.7899 | 0.8045 | 1 | 0.7783 | 0.6509 | 0.9312 | 0.578 | 0.5099 | 0.8419 |
|  | p-value | 0 | 0 | 0 | 0 | 0 | 0 | 0.0002 | 0 |  |
|  | n | 48 | 48 | 48 | 48 | 48 | 48 | 48 | 48 | 48 |
| LB | coef | 0.7105 | 0.7298 | 0.7783 | 1 | 0.7557 | 0.7592 | 0.6909 | 0.6676 | 0.8494 |
|  | p-value | 0 | 0 | 0 | 0 | 0 | 0 | 0 | 0 |  |
|  | n | 48 | 48 | 48 | 48 | 48 | 48 | 48 | 48 | 48 |
| SMLW | coef | 0.5695 | 0.5217 | 0.6509 | 0.7557 | 1 | 0.6599 | 0.4136 | 0.8304 | 0.8214 |
|  | p-value | 0 | 0.0001 | 0 | 0 | 0 | 0.0035 | 0 | 0 |  |
|  | n | 48 | 48 | 48 | 48 | 48 | 48 | 48 | 48 | 48 |
| LLL | coef | 0.7972 | 0.8014 | 0.9312 | 0.7592 | 0.6599 | 1 | 0.6177 | 0.4469 | 0.8531 |
|  | p-value | 0 | 0 | 0 | 0 | 0 | 0 | 0.0015 | 0 |  |
|  | n | 48 | 48 | 48 | 48 | 48 | 48 | 48 | 48 | 48 |
| LLB | coef | 0.4657 | 0.6068 | 0.578 | 0.6909 | 0.4136 | 0.6177 | 1 | 0.3964 | 0.5805 |
|  | p-value | 0.0009 | 0 | 0 | 0 | 0.0035 | 0 | 0.0053 | 0 |  |
|  | n | 48 | 48 | 48 | 48 | 48 | 48 | 48 | 48 | 48 |
| LLW | coef | 0.4161 | 0.4655 | 0.5099 | 0.6676 | 0.8304 | 0.4469 | 0.3964 | 1 | 0.6988 |
|  | p-value | 0.0033 | 0.0009 | 0.0002 | 0 | 0 | 0.0015 | 0.0053 | 0 |  |
|  | n | 48 | 48 | 48 | 48 | 48 | 48 | 48 | 48 | 48 |
| TLWP | coef | 0.8263 | 0.8866 | 0.8419 | 0.8494 | 0.8214 | 0.8531 | 0.5805 | 0.6988 | 1 |
|  | p-value | 0 | 0 | 0 | 0 | 0 | 0 | 0 | 0 |  |
|  | n | 48 | 48 | 48 | 48 | 48 | 48 | 48 | 48 | 48 |

CORRELATION ANALYSIS

Pearson's product-moment correlation, Prob > |r|

**Keys to abbreviations**; **PH**: Plant height, **NL**: Number of leaves, **LL**: Leaf length, **LB**: Leaf breadth, **LL**L: Largest leaf length, **LLB**: Largest leaf breadth, **SMLW**: Single mature leaf weight, **LLW**: Largest leaf weight, **TLWP**: Total leaf weight per plant,
