# Supplementary figures and images for: Dataset on the influence of zinc foliar application and vermicompost on agromorphogenic traits of Aloe vera
Source: Data Brief. 2021 Sep 30;38:107436. doi: 10.1016/j.dib.2021.107436 (PMC8498230; doi:10.1016/j.dib.2021.107436)

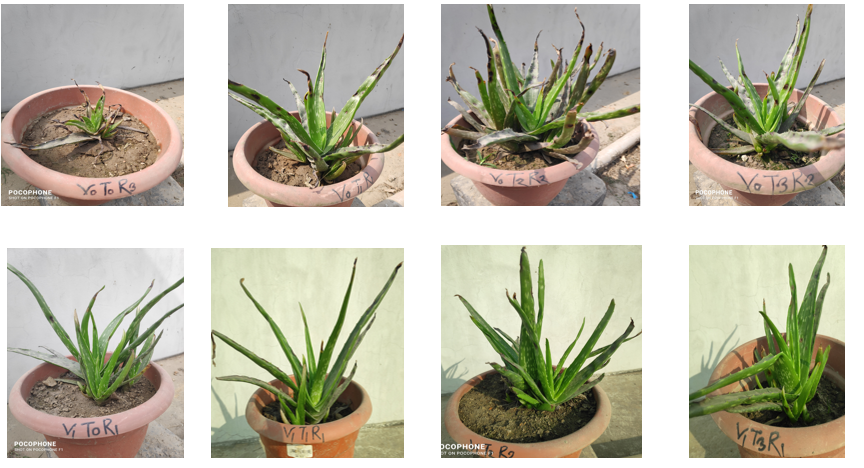


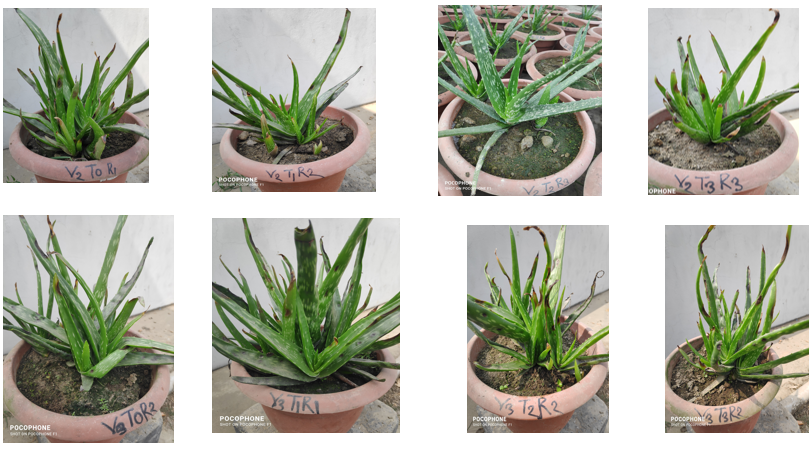


Plate: Phenotypic variation under different treatment ( 60 days old aged plants)

Supplement: Supplementary file 20 [file mmc20.zip › Image of aloe vera under experiment.docx]
